# Clinical profile and outcome of road traffic accidents among adult patients treated at urban government hospitals in Ethiopia

**DOI:** 10.1016/j.afjem.2026.100997

**Published:** 2026-08-01

**Authors:** Ayenew Amare, Rediet Getu, Bitania Debalkew, Mihretu Jegnie, Aklilu Azazh

**Affiliations:** a Department of Emergency Medicine, College of Health Sciences, Addis Ababa University, Addis Ababa, Ethiopia; b Biomedical Sciences Education and Service Directorate, College of Health Sciences, Debre Tabor University, Debre Tabor, Ethiopia

**Keywords:** Road traffic accidents, Clinical profile, Outcome, Emergency care, Ethiopia

## Abstract

**Background:**

Around 1.19 million people per year die as a result of traffic collisions worldwide. Road traffic injuries (RTI) are on the decline in industrialized nations but rising in many low and middle income countries, including Ethiopia. However, there is a lack of evidence regarding the clinical profile and outcome of RTI in Ethiopia. This study assessed the clinical profile, emergency department (ED) visit outcome, and factors associated with ED mortality of RTI victims treated at five selected government hospitals in Addis Ababa.

**Methods:**

An institutional-based prospective cross-sectional study was conducted by interviewing RTI victims and attendants as well as reviewing their charts in July 2023. Systematic random sampling was employed to select 247 samples. The primary outcome measure was ED mortality. Secondary outcome measures included other ED treatment outcomes (treated and discharged, hospital admission, ICU admission, and referral).

**Result:**

A total of 224 RTI victims were included in this study. The male-to-female ratio was 2.07:1, with the 24–33 age group predominantly affected by trauma. Ambulance was the predominant mode of arrival to the hospital (89.7%). Mostly pedestrians were affected, 161 (71.9%). Although the largest group of injured patients (94, 42.0%) were treated and immediately discharged from the ED, a significant number (45, 20.1%) required intensive care unit admission. ED RTI mortality rate was 1.8%. Delayed arrival (*p* = 0.02), lack of pre-hospital care (*p* = 0.01), and poly-trauma (*p* = 0.02) were associated with increased ED death.

**Conclusion:**

This study revealed that RTIs primarily affect young, economically engaged men and pedestrians. The overall mortality in the ED was low. Delayed arrival (1–24 h), lack of pre-hospital care, and poly-trauma were associated with increased ED death. Various interventions are suggested from improving prehospital emergency care, to improving tertiary hospital trauma care, as well as prevention strategies including promoting public awareness of RTI dangers.

## African relevance

• Traffic injuries severely burden young, productive populations across Africa.
• Pedestrians represent a massive, vulnerable safety gap in African urban centers.
• Pre-hospital care deficits and delayed center arrival drive trauma mortality.
• Africa needs coordinated emergency systems for uniform pre-hospital stability.
• Prioritizing rapid trauma intervention is vital to reduce emergency deaths.

## Introduction

Approximately 1.19 million people die due to road traffic accidents (RTAs) every year, which makes RTAs a severe public health problem worldwide. This accounts for a daily fatality rate of >3200 people [1]. Apart from the reported traffic-related fatalities, RTAs are projected to result in as many as 20 to 50 million non-fatal injuries annually [2]. The leading cause of Disability-Adjusted Life Years among adolescents and young adults, aged 10–24 and the prime working age group of 25–49, is due to road injuries, as stated in the Global Burden of Disease Study 2019 [3]. This enormous burden is estimated to cause a reduction of about 3% in global gross domestic product and as high as 5% in low- and middle-income countries (LMIC) [1]. The difference in RTA patterns of high and LMIC highlights the most striking trend in both populations. While the share of injury burden is increasing rapidly in LMIC, RTA-related fatalities continue to decline in high income countries [[2], [3]].

This is especially true in Africa, where, despite Europe’s significantly higher vehicle density, the recorded death rate from RTAs is the highest in the world (26.6 per 100,000 people), compared to 9.3 per 100,000 in Europe [4]. Today, road traffic collisions (RTC) are one of the leading causes of injury, disability, and death in Ethiopia, making it a major and continually increasing threat to the national transportation infrastructure [2]. The World Health Organization (WHO) estimated that the fatality rate of RTA in Ethiopia was 25.3 per 100,000 in 2015, which was much higher than rates seen in developed countries [1]. Addis Ababa is the most densely populated city of the country, and it also shares the highest number of RTA fatalities and injuries. RTAs were responsible for 27% of all emergency visits, 5% of hospitalizations, and 3% of city fatalities, according to data from the Health Sector Development Program IV (2010/11 to 2014/15) [5].

Despite the alarming rate of RTAs in Ethiopia, comprehensive and contemporary data on the country’s RTC profile remains scarce [2,[4], [5], [6]]. To the best of the researchers’ knowledge, no recent multi-site study has thoroughly examined the relationship between specific risk factors and the mortality outcome of RTA victims referred to trauma centers. Therefore, the purpose of this study is to evaluate the clinical profile, patient outcome, and the specific factors influencing ED mortality of RTC victims at five selected government hospital EDs and trauma centers in Addis Ababa.

## Methods and materials

### Study setting

The study was conducted in five selected public hospitals in Addis Ababa that provide trauma care at national level. These selected public hospitals are Tikur Anbesa Specialized Hospital, Addis Ababa Burn, Emergency & Trauma Hospital (AaBET), Zewuditu Memorial Hospital, Menelik II Comprehensive Specialized Hospital, and All African Leprosy Tuberculosis Rehabilitation and Training (ALERT) Hospital. An institutional based prospective cross-sectional study was conducted by interviewing patients and attendants as well as reviewing their charts presenting with RTA injuries to the five selected public hospitals from July 1–30/2023.

The source population was all RTA patients at five selected government trauma centers. The study population were all adult RTA patients treated at five selected governmental trauma center that fulfill the inclusion criteria during the study period. All RTA adult patients (age greater than or equal to 15 year) treated at five selected government hospitals during the study period were eligible for this study. Non-traumatic patients visiting the ED were excluded. Injuries by non-motorized vehicles like bicycles and carts were also excluded to ensure a sample focused on high-kinetic energy motor vehicle collisions. Incomplete records were excluded, defined as any form missing the ED visit outcome or critical clinical parameters that could not be retrieved from the concurrent medical charts.

The required sample size was calculated using the prevalence of RTA taken from a previous study conducted in AaBET Hospital (*P* = 0.364), giving a final sample size of 247.

A systematic random sampling technique was employed prospectively to select study participants as they presented to the EDs. The average monthly RTA flow in the last 12 months was estimated to be 604 patients based on hospital registries. The sampling interval (K) was determined by dividing the population size, N = 604, by the required sample size, n = 247 (k = 604/247 = 2.45∼2). As a result, during the data collection period (July 1–30, 2023), every 2nd eligible RTA patient presenting to the respective EDs was sequentially selected. The first participant at each hospital was selected using a lottery method at the beginning of the study period.

### Operational definitions

RTA: Any instance of physical injury or damage to the body or body part collides with moving vehicle; ED mortality: A death of an RTA patient strictly within the ED after arriving at the hospital with vital signs present. It excludes patients who were Brought In Dead with no signs of life upon arrival, as well as those who were admitted to inpatient wards or the ICU and subsequently passed away.; Economic activity: Individuals within a defined age range of 15 to 64 years, considered economically active and available to work [7].; Alcohol consumption: Labeled as consumed based on self-report and ascertained by triangulating with proxy interviews and clinical observation.

### Data collection and data quality assurance

A pre-tested, structured questionnaire and data abstraction form was utilized which was carefully modified from the WHO Injury Surveillance Guideline [8]. The questionnaire was initially prepared in English and then translated into the local language, Amharic. It was subsequently back-translated to English by two independent language experts fluent in both languages to maintain consistency. The questionnaire is composed of three parts (socio-demographic characteristics, clinical profile of patients, and EDs visit outcomes). Data were collected using a combination of methods: through interviews and a concurrent patients’ medical records/charts abstraction. Socio-demographic and mechanism of injury data were collected via face to face interview; clinical findings and ED outcomes were abstracted from medical charts. Direct face-to-face interviews were conducted with 175 (78.1%) conscious and stable patients. In the remaining 49 (21.9%) cases where severe injuries or immediate mortality limited a direct interview, a proxy protocol was followed to interview family members, accompanying attendants, or traffic police. To protect data reliability and eliminate recall or information bias, proxy interviews were strictly limited to basic socio-demographic and crash mechanism variables. To minimize recall bias regarding the exact time of the incident, a cross-verification protocol was implemented. The time of the crash was primarily abstracted from objective external sources, including traffic police logs, EMS ambulance records, and formal referral letters. For patients who were deceased or in severe distress, this information was obtained from proxy respondents (accompanying attendants or police officers).

A team of five data collectors (BSc Nurses) and one supervisor (Orthopedic resident) were recruited for the data collection and supervision, respectively. A two days training was given to the data collectors and supervisors. During data collection, regular supervision and follow up was made. Pretest was done in a public hospital which was not selected for the main study data collection on 5% of the sample size.

### Data analysis

Data were entered and analyzed using Statistical Package for the Social Sciences (SPSS) (IBM, Armonk, USA) version 26. The primary outcome was ED mortality. Secondary outcomes included ED outcomes categorized as discharged, ward admission, ICU admission, and referral. Exposure variables included type of vehicle, interval between injury and arrival, alcohol use, anatomical site of injury, and pre-hospital care given. The association between the dependent (mortality at ED) and independent variables was assessed using Fisher’s Exact Test. P-value of <0.05 was considered statistically significant.

### Ethical consideration

Ethical clearance was obtained from the Ethical Review Committee of the Addis Ababa University College of Health Science’s Emergency Medicine Department (Ref: EM/59/2023). Permission was obtained from the selected five public hospitals through a formal letter of cooperation, and patient confidentiality were held anonymously. Informed consent was obtained from all the participants who were greater than or equal to 18 and for ages 15 to 17 assent plus parental consent was taken. All information was kept confidential, and the organization ensured that no information was shared with any third parties or institutions without their permission.

## Results

A total of 224 RTI victims were included in this study across the adult EDs of the five government hospitals in Addis Ababa, yielding a response rate of 90.7%. This final sample excludes 23 incomplete records out of the initial 247 sampled cases. The majority of the RTA victims included in this study were young, economically active males (Table 1).Of the RTA casualties, 201 (89.7%) lived in urban areas. The majority of these patients 159 (71%) were Addis Ababa city dwellers.

**Table 1:** Socio-demographic characteristics of patients visiting five government hospitals emergency departments, Addis Ababa, 2023.

Variables with categories		Frequency	Percent
Sex	Male	151	67.4
	Female	73	32.6
Age	15–23	17	7.6
	24–33	104	46.4
	34–43	63	28.1
	44–53	24	10.7
	54–64	15	6.7
	>64	1	0.4
Marital status	Single	112	50
	Married	109	48.7
	Divorced	1	0.4
	Widowed	2	0.9
Place of residency	Urban	201	89.7
	Rural	23	10.3
Regions of referral	Addis Ababa	159	71
	Oromia	50	22.3
	Amhara	7	3.1
	Afar	6	2.7
	SNNP	1	0.4
	Others	1	0.4

The majority of patients 159 (71.0%) reached the hospital between 1- and 24-hours post-injury. Only 18 (8.0%) RTI victims arrived within the first hour of the accident. Most accidents occurred during the afternoon time, specifically between 12:00 PM and 06:‍00 PM, accounting for 65 (28.3%) of all cases. The majority of patients (201, 89.7%) were transportedby ambulance, followed by those using a private car (13, 5.8%), and‍ taxi (9, 4.0%). Only 34 (15.1%) of RTI patients received pre-hospital treatment. Pedestrians were by far the most frequent victims of RTAs 169 (71.9%). The vehicles most commonly involved in these accidents were minibuses (108, 48.2%), followed by heavy goods vehicles (49, 21.9%) (Table 2).

**Table 2:** Pre-hospital characteristics and presentation of road traffic injury patients visiting five governmental hospital trauma center emergency departments, Addis Ababa, 2023.

Variables with categories		Frequency	Percent
Interval between injury and arrival to tertiary hospital	<1hr	18	8
	1–24hr	159	71
	>24hr	47	21
Distance from injury site to tertiary hospital	0 – 15 km	55	24.5
	16 – 35 km	100	44.7
	> 35 km	69	30.8
Referred from	Health center	128	57.1
	Hospital	66	29.5
	Private facilities	27	12.1
Modes of transportation	Ambulance	201	89.7
	Taxi	9	4
	Private car	13	5.8
	Walking	1	0.4
Pre hospital care given	Yes	34	15.1%
	No	190	84.9%
Alcohol or substance use at time of injury	Yes	58	25.9
	No	166	74.1
Trauma victims	Pedestrian	161	71.9
	Passenger	29	12.9
	Driver	31	13.8
	Motor cyclist	3	1.3
Type of vehicle	Mini bus	108	48.2
	Heavy good car	49	21.9
	Taxi	48	21.4
	Tricycle	11	4.9
	Motorcycle	6	2.7
	Others	2	0.9

**Fig. 1:**
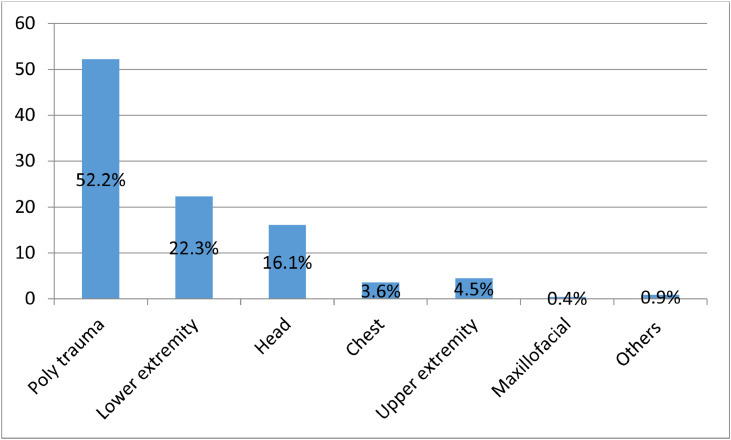
Anatomical Site of Injury of Road Traffic Injury Patients Visiting Five Governmental Hospital Trauma Center Emergency Departments, Addis Ababa, 2023.

The most prevalent pattern of injury was poly-trauma (117, 52.9%). Among specific anatomical sites, the lower extremity was the next most commonly injured region (50, 22.3%), followed by the head (36, 16.1%)(Fig. 1).

**Fig. 2:**
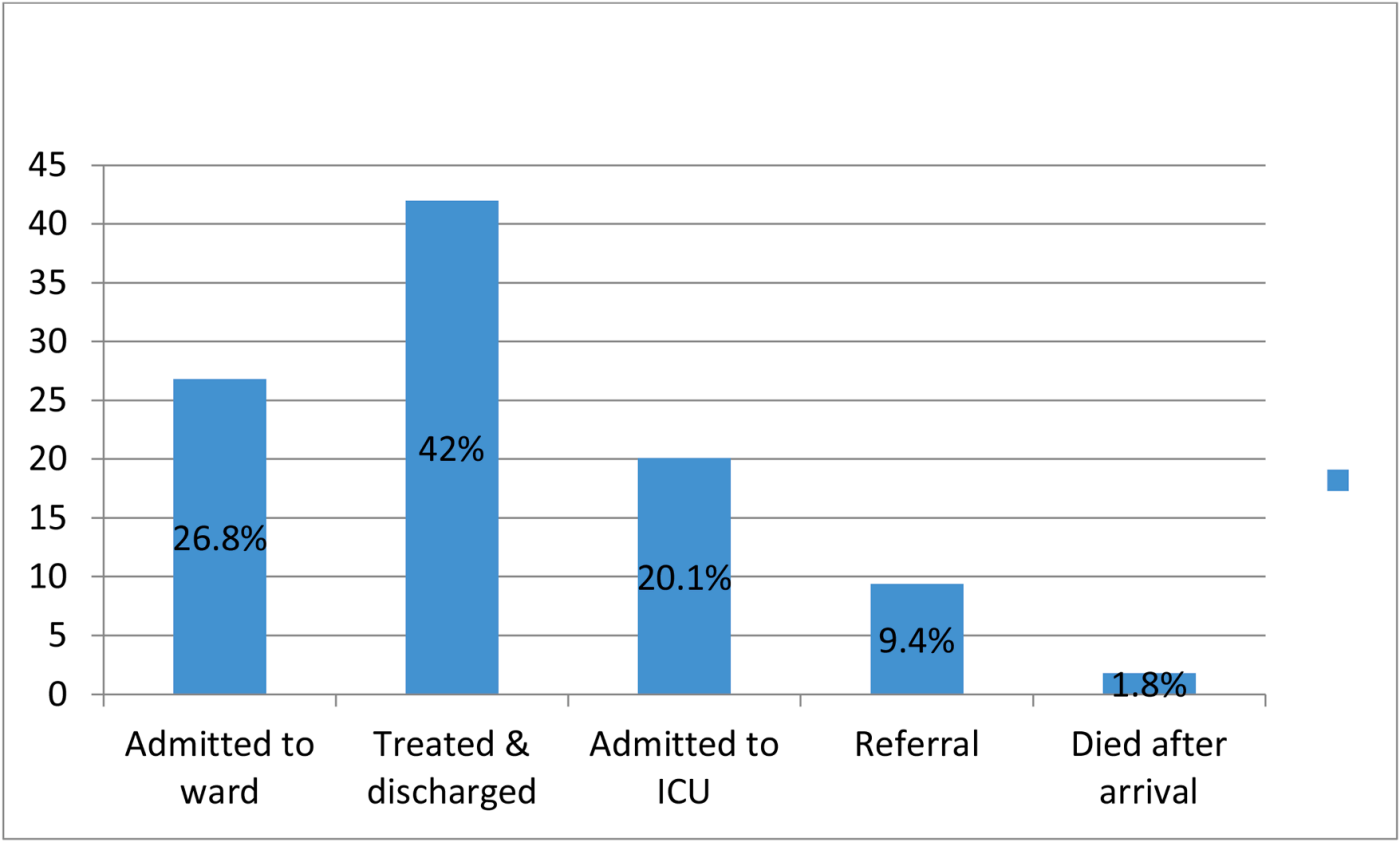
Emergency Visit Outcome of Road Traffic Injury Patients Visiting Five Governmental Hospital Trauma Center Emergency Departments, Addis Ababa, 2023.

The largest group of injured patients (94, 42.0%) were treated and immediately discharged from the ED (Fig. 2). Tragically, 4 (1.8%) of the injured patients died after arriving at the ED.

Fisher’s exact test was utilized to assess the association between key clinical factors and mortality within the ED (Table 3). The interval between injury and arrival was significantly associated with mortality (*P* = 0.02), with patients arriving at the hospital between 1 and 24 h post-injury accounting for 3 (1.3%) of the non-survivors, thus indicating a higher mortality risk compared to those arriving earlier or later. Polytrauma as an anatomical site of injury was also significantly associated with mortality (*P* = 0.02). Furthermore, the lack of pre-hospital care given showed a statistically significant association with mortality (*P* = 0.01). Specifically, 3 (1.3%) of the non-survivors were patients who did not receive any pre-hospital care.

**Table 3:** Effect of patients’ clinical profile on emergency road traffic accident mortality, Addis Ababa, 2023.

Variables with categories		Mortality		Total	P-value
		Survivors	Non-survivors		
Type of vehicle	Mini bus	105 (46.9)	3 (1.3)	108 (48.2)	0.87
	Heavy good car	48 (21.4)	1 (0.4)	49 (21.9)	
	Taxi	48 (21.4)	0 (0)	48 (21.4)	
	Bajaj	11 (4.9)	0 (0)	11 (4.9)	
	Motor cycle	6 (2.7)	0 (0)	6 (2.7)	
	Others	2 (0.9)	0 (0)	2 (0.9)	
Interval between injury & arrival	<1hr	17 (7.6)	1 (0.4)	18 (8)	0.02*
	1–24hr	156 (69.6)	3 (1.3)	159 (71)	
	>24hr	47 (21)	0 (0)	47 (21)	
Alcohol use	Yes	57 (25.4)	1 (0.4)	58 (25.9)	0.23
	No	163 (72)	3 (1.3)	166 (74.1)	
Anatomical site of injury	Poly trauma	114 (50.9)	3 (1.3)	117 (52.2)	0.02*
	Lower extremity	50 (22.3)	0 (0)	50 (22.3)	
	Head	36 (16.1)	0 (0)	36 (16.1)	
	Chest	7 (3.1)	1 (0.4)	8 (3.6)	
	Upper extremity	10 (4.5)	0 (0)	10 (4.5)	
	Maxillofacial	1 (0.4)	0 (0)	1 (0.4)	
	Others	2 (0.9)	0 (0)	2 (0.9)	
Pre-hospital care given	Yes	33 (14.7)	1 (0.4)	34 (15.1)	0.01*
	No	187 (85.3)	3 (1.3)	190 (84.8)	

⁎ Statistically significant.

## Discussion

Road traffic accidents (RTAs) in Ethiopia have increased, leading to a considerable number of fatalities, serious injuries, and socioeconomic effects. Some of the factors contributing to this trend include rapid urbanization, weak enforcement of laws relating to traffic, inadequate road infrastructure, and lack of comprehensive safety measures. The majority of RTA victims (46.4%) fell within the 24–33 age group, which aligns with previous similar studies in Ethiopia [9,10]. Similarly, males (67.4%) were more affected by RTAs than females (32.0%). This higher male prevalence is a consistent finding across multiple national and international RTA studies [[9], [10], [11]], likely reflecting higher male exposure to traffic and road use.

Road traffic injury was more common among pedestrians 161 (71.9%), drivers 31 (13.8%), and passengers 29 (12.9%). This result was consistent with other research from LMIC nations as well as earlier research carried out in Ethiopia [12,13]. This may be the result of poorly designed roads, insufficient pedestrian-friendly sidewalks, and traffic signals nationwide. It might also be the result of a lack of public awareness of traffic laws, rude driving and motorist conduct, and countrywide traffic law violations by pedestrians and drivers [13].

The majority of collisions occurred in the afternoon throughout the day, 65 (28.3%). This result was in line with the results of prior research [9,13,14]. Additionally, 201 (89.7%) collisions happened in urban areas. This result was not consistent with study done at Dilla Teaching Hospital [15]. There may be more collisions in metropolitan locations and throughout the day due to factors like mixed traffic flow systems, poor road networks, and traffic jams during the day [13].

Regarding the mode of transport, our survey found that the vast majority of patients (89.7%) arrived at the hospital via ambulance, with only 9.8% being driven by taxi or private vehicle. This contrasts sharply with a previous study conducted at Tikur Anbessa Specialized Teaching Hospital, where a large majority of RTI victims (35.1%) reportedly used minibuses as their mode of transportation [13]. This difference may suggest a recent improvement or change in patient retrieval methods, or it could reflect the higher capacity and more centralized ambulance services servicing the five major trauma centers included in the current study. Furthermore, the greater use of ambulances is likely influenced by the growing involvement of private entities, such as Tebita Ambulance, in providing and expanding pre-hospital services in Addis Ababa. This trend is consistent with similar observations at other major trauma centers like AaBET Hospital [10].

About 34 (15.1%) casualties, received pre-hospital care. This result was greater than that of earlier research in Tanzania, which is reported to 0% [16] and Ethiopia which is reported to 14.3% [13]. The development of organized pre-hospital services in Addis Ababa and the involvement of private business groups in the ambulance and the pre-hospital services, such as Tebita Ambulance in Addis Ababa, may be the cause of the higher ambulance utilizations and pre-hospital services received by victims in this study.

After an injury, there is a critical window of time during which life-sustaining treatment should be started. Prompt management begins during this window of opportunity, giving the victim the best chance of survival and reducing the likelihood of subsequent problems leading to death. According to this study, the majority of the 159 (71%) victims of traffic accidents arrived at the first medical facility between one and twenty-four hours.

The overall ED mortality rate was 1.8% (4 non-survivors out of 224 patients). This observed rate is slightly higher than figures reported in some international settings, such as India (1.0%) [11], Canada (1.2%) [17], and Korea (0.6%) [18], and Ethiopia (1.47%) [13]. Conversely, the rate is significantly lower than figures found in some regional studies, including sub-Saharan Africa (4.2%) [12] and Dilla Hospital in Ethiopia (6.0%) [15]. These wide discrepancies in mortality outcomes can be attributed to several factors inherent to the study setting and study sample and inclusion criteria; In the current study, a significant proportion of patients were referred from other health facilities, and those presenting after 24 h were likely already resuscitated and stabilized elsewhere. This inherent referral pattern may, therefore, lead to an underestimation of the true immediate ED mortality rate for all RTA victims in the centers.

A striking finding in this study was the statistically significant association between lack of pre-hospital care and mortality. The study indicated that patients not treated with pre-hospital care constituted the majority of non-survivors. This suggests a deficiency at the systemic level in Addis Ababa’s EMS infrastructure. Lacking initial stabilization-such as airway management and bleeding control-likely contributes to preventable deaths prior to the patient even arriving at the hospital. This finding is consistent with studies from other developing nations where the lack of organized ambulance systems stands out as a major independent predictor of trauma mortality [[19], [20], [21], [22], [23]].

Another significant association with mortality of RTI victims was observed with time interval from injury to arrival. There was a significantly higher risk of death for patients arriving between 1- and 24-hours post-injury compared with those arriving within the first hour. This result reinforces the long-established concept of the "Golden Hour" in trauma care, in which early resuscitation dramatically improves survival chances. The delay in arrival in our setting may be attributed to traffic congestion in Addis Ababa or reliance on bystanders for transportation [[20], [21], [22], [23]] Surprisingly, patients who presented after 24 h did not demonstrate the same high risk for mortality. This finding is most likely to be explained by the referral nature of the subjects studied rather than the severity of their injury. Most of these late-presenting patients were referred from other health facilities where they were already resuscitated and stabilized. Thus, the most critical window for immediate mortality would have elapsed under medical supervision elsewhere, presenting in a more clinically stable state upon arrival in the study hospitals [24].

The anatomical site of injury also contributed to the pattern of patient outcome. Poly-trauma was associated with mortality. This is a biologically plausible observation, given that patients with multisystem injuries usually have more physiological derangement and a cumulatively higher risk of shock and multi-organ failure compared to their counterparts with isolated injuries [25]. The care for poly-trauma patients is multidisciplinary and resource-intensive, which can stress the capacity of government hospitals and may affect outcomes [26].

*Limitations*

The study was confined to five selected major government trauma centers in Addis Ababa. Thus, the profile of injuries and reported outcomes might not be fully generalizable to the whole country or to RTA victims treated in smaller or private facilities within the capital. Most critically, the small number of mortality outcomes observed-only four non-survivors-considerably limited the depth of statistical analysis. This limitation precluded the use of a robust multivariable analysis, such as binary logistic regression, and the analyses were restricted to simple bivariate tests (Fisher's Exact Test). As such, although the study determined statistically significant associations of mortality with factors such as lack of pre-hospital care, late arrival, and poly-trauma, the results cannot definitely establish these factors as independent predictors of death. The high proportion of RTA in Addis Ababa might be due to geographic selection bias as the study was conducted in hospitals found in Addis Ababa only. Another limitation of this study is that alcohol consumption was determined through a combination of self-reporting, proxy interviews, and clinical observation rather than objective blood alcohol concentration or breathalyzer testing.

## Conclusions

RTA in this study setting disproportionately affected young males and pedestrians. While the study revealed a relatively low road traffic accident related mortality at the ED, delayed arrival at tertiary treatment centers, lack of pre-hospital care, and sustaining poly-trauma were associated with increased likelihood of emergency department death. Even though there is an encouraging, emerging trend toward the use of ambulances, the overall high risk associated with delayed arrival reinforces the need for rapid trauma intervention. To reduce mortality related to road traffic accident, a centrally coordinated, citywide emergency medical service system must be established to ensure uniform pre-hospital stabilization. Furthermore, tertiary treatment centers should implement fast-track triage protocols specifically tailored for poly-trauma patients arriving within the critical 24-hour window to minimize diagnostic delays. To address the high pedestrian toll, municipal authorities must prioritize targeted infrastructural upgrades, such as designated pedestrian walkways, elevated zebra crossings, and improved street lighting in high-risk urban zones. Targeted and continuous public awareness campaigns focused on vulnerable road users should also be implemented.

## CRediT author contributions

**AA**: Conceptualization, Data curation, Formal analysis, Investigation, writing -original draft, **RG**: Conceptualization, supervision, data curation, Writing – review & editing. **BD**: Supervision, data curation, Writing – review & editing. **MJ**: Data curation, Writing- review & editing, Investigation, formal analysis. **AA**: Conceptualization, supervision, Writing – review & editing, Investigation, formal analysis. All authors approved the version to be published and agreed to be accountable for all aspects of the work.

## Dissemination of results

Results from this research study were shared with staff members of the ED through thesis presentation discussion.

## Funding declarations

This research received no specific grant from any funding agency in the public, commercial, or not-for-profit sectors.

## Declaration of competing interest

The authors declare that they have no known competing financial interests or personal relationships that could have appeared to influence the work reported in this paper.
